# Precision treatment of gastrointestinal tumours and liver disease interaction mechanisms based on multi-omics data and microbiome hubs

**DOI:** 10.3389/fcimb.2026.1791531

**Published:** 2026-03-13

**Authors:** Bangxing Lin, Shizheng Tong, Chaokai Ba, Xiang Wang

**Affiliations:** 1 The Fourth School of Clinical Medicine, Zhejiang Chinese Medical University, Hangzhou First People’s Hospital, Hangzhou, Zhejiang, China; 2 Zhejiang Key Laboratory of Zero Magnetic Medicine, School of Medicine, Affiliated Hangzhou First People’s Hospital, Westlake University, Hangzhou, Zhejiang, China

**Keywords:** artificial intelligence, gastrointestinal tumour, gut microbiota, liver disease, multi-omics integration, single-cell sequencing

## Abstract

The global prevalence of gastrointestinal tumours and the bottlenecks in their diagnosis and treatment are being systematically overcome by the multi-omics revolution: high-throughput technologies are driving the multidimensional integration of genomics-transcriptomics-proteomics-metabolomics to comprehensively decode the genetic architecture of tumours. Meanwhile, the gut microbiota, acting as a core regulatory hub, drives carcinogenesis through immune microenvironment remodelling and metabolic pathway hijacking, further facilitating proteome-metabolome multidimensional integration, comprehensively decoding tumour genetic architecture. The gut microbiota, acting as a core regulatory hub, drives carcinogenesis through immune microenvironment remodelling and metabolic pathway hijacking, while mediating a vicious cycle network linking liver disease and tumours via the gut-liver axis. This review examines the application of multi-omics technologies in gastrointestinal tumour research, summarises the role of gut microbiota in tumourigenesis and its interaction with liver disease, and envisions future interventions targeting the gut microbiome for early disease diagnosis and precision treatment.

## 1 Introduction

Gastrointestinal tumours rank among the most prevalent cancers globally, though their incidence and mortality rates vary significantly by region (Li S. et al., 2023; Bray et al., 2024). Despite recent advances in early detection and treatment, the marked heterogeneity of these tumours poses substantial challenges for subsequent therapeutic and diagnostic approaches (Karimi et al., 2014; Tan and Yeoh, 2015). Endoscopic resection and conventional chemotherapy remain traditional treatment modalities, yet their impact on patient prognosis remains limited (Karimi et al., 2014). However, with continuous advancements in sample processing techniques, high-throughput instrumentation, and computational analysis capabilities, omics technologies have experienced rapid development (Wang et al., 2014). The application of omics technologies enables comprehensive elucidation of the genetic and molecular characteristics of gastrointestinal tumours (Xu et al., 2017; Li S. et al., 2023).

Through multi-omics analysis of samples from gastrointestinal tumour patients, researchers have discovered that cancerous tissues from different individuals exhibit distinct biological and molecular characteristics of gastrointestinal tumours (Castaño-Vinyals et al., 2015). Genomic stratification, which links specific targeted therapies to particular gene mutations, provides the theoretical foundation for subsequent personalised treatments, such as molecular therapy targeting genetic markers (Wang et al., 2014; Tan and Yeoh, 2015; Sohn et al., 2017). Translational omics analysis reveals the relationship between targeted gene molecules and cancer progression (Kim et al., 2011; Xu et al., 2017). By identifying distinct subtypes and molecular markers through proteomic profiling, which integrates abnormally expressed proteins in tumours with molecular signalling networks, this provides novel therapeutic targets for treatment (Cristescu et al., 2015). Concurrent metabolomics studies characterise metabolic pathways in gastrointestinal and liver cancers through patient biofluids, analysing post-treatment metabolite signatures to predict clinical outcomes (Kang et al., 2016). Single-cell sequencing and spatial transcriptomics techniques analyse cellular spatial organisation within tumours and their microenvironments, enabling researchers to study gene expression and cell-cell interactions at single-cell resolution (Krug et al., 2020; Wang et al., 2024).

**Figure 1:**
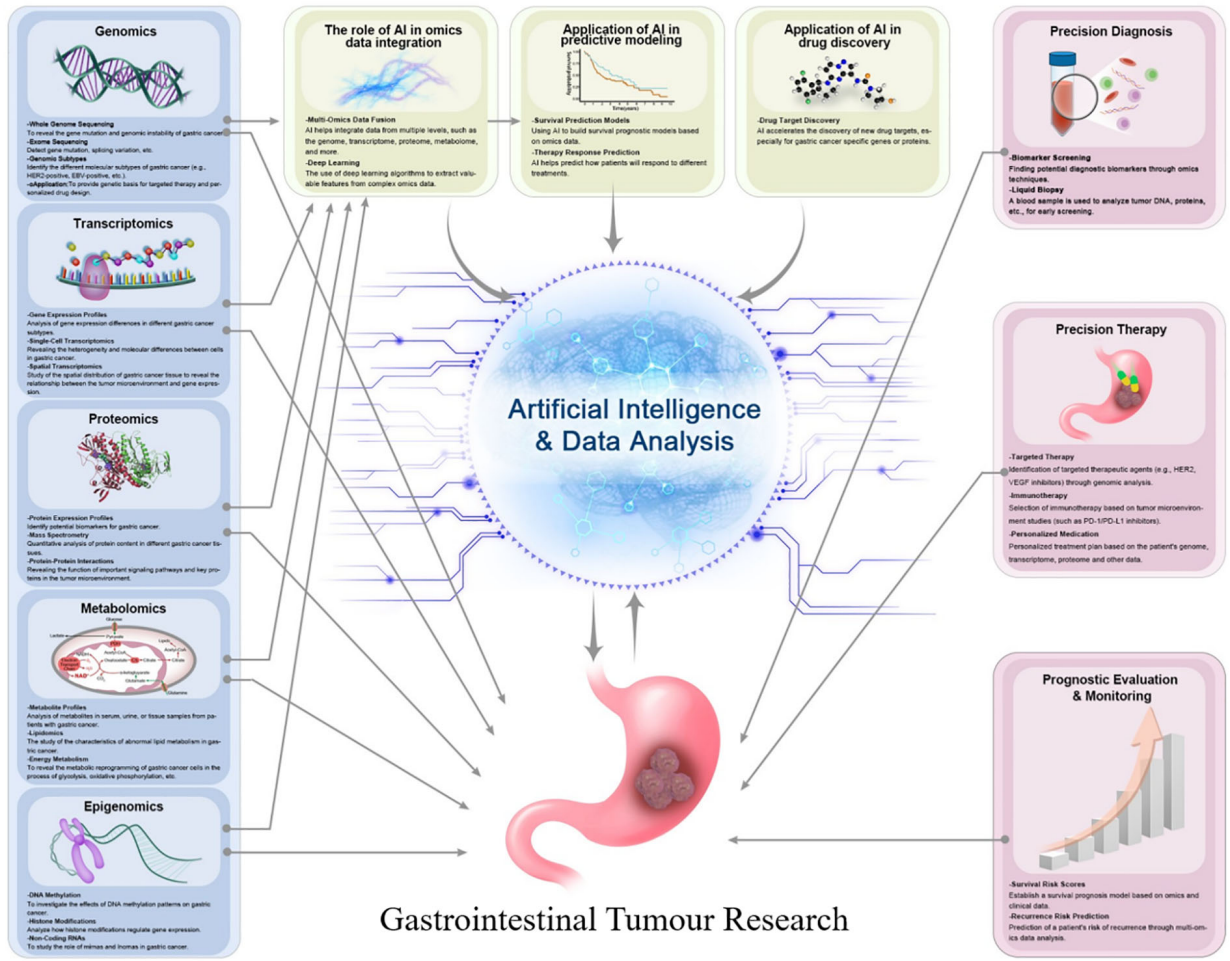
An integrated multi-omics framework for AI-driven gastric cancer research.

This review summarises the application of multiple omics under artificial intelligence for in-depth analysis of gastrointestinal tumours, thereby elucidating disease relationships at the molecular level. **Figure 1** illustrates omics applications in gastrointestinal tumours, encompassing genomics, transcriptomics, proteomics, metabolomics, and epigenomics. Through big data analysis, it deepens understanding of the biological mechanisms underlying gastrointestinal tumours, providing a theoretical foundation for subsequent precision therapies.

Gut microbiota dysbiosis is emerging as a core driver in the initiation and progression of gastrointestinal tumours: it reshapes the immune microenvironment—where chronic inflammation induced by specific pathogens drives immune editing processes, establishing a pathological foundation for carcinogenesis; metabolic byproducts (such as SCFAs/bile acids) dual-regulate mucosal barrier integrity and oncogenic pathways (e.g., the p53 signalling network); furthermore, direct microbiota-epithelial cell interactions reshape tumour phenotypes (Vucic et al., 2012). Collectively, these discoveries propel targeted microbial interventions to the forefront, establishing a new paradigm for early screening and precision treatment in gastrointestinal oncology.

Gastrointestinal (GI) tumours frequently coexist with chronic liver diseases, including metabolic-associated fatty liver disease (MAFLD/NAFLD), viral hepatitis, alcoholic liver disease, cholestatic liver disease, and cirrhosis. This comorbidity warrants clinical attention, as hepatic dysfunction and portal hypertension may: (i) remodel systemic and mucosal immunity; (ii) compromise intestinal barrier integrity and increase bacterial translocation; (iii) alter bile acid and exogenous substance metabolism; (iv) limit the feasibility and safety range of antitumour therapies (e.g., cytotoxic chemotherapy, targeted agents, and immunotherapy). Conversely, gastrointestinal tumours can exacerbate liver injury through systemic inflammation, malnutrition/sarcopenia, drug-induced liver injury, and critically, liver metastases, forming a bidirectional pathogenic cycle. Therefore, a complication-oriented framework is needed that integrates tumour biology with liver disease severity, treatment tolerance, and the dynamic microbiome-metabolism state along the gut-liver axis to enable clinically actionable stratification and precision interventions.

The interaction network between gastrointestinal tumours and liver diseases reveals a triple pathological axis: immune-metabolic microenvironment remodelling driven by chronic liver diseases (such as cirrhosis) disrupts gastric mucosal barrier homeostasis through inflammatory mediator diffusion and toxin accumulation, significantly elevating gastrointestinal tumour risk; conversely, liver metastasis from gastrointestinal tumours forms a vicious cycle accelerating hepatic functional failure. where gut microbiota serve as a pivotal regulatory hub. Via the gut-liver axis, they mediate immune reprogramming and metabolic toxin transfer, bidirectionally driving pathological processes in both organs. This discovery establishes a molecular foundation for multi-level intervention strategies targeting the microbiota.

This review decodes frontier advances in gastrointestinal tumour genomics, focusing on the gut microbiota’s pivotal regulatory role in tumour initiation and progression, alongside its pathological axis interactions with liver disease. Through multi-omics integration strategies, it unveils microbiota-mediated molecular cascade mechanisms, ultimately establishing a paradigm shift towards early diagnosis and precision therapy targeting the microbiome.

## 2 Literature review

### 2.1 Current landscape and emerging trends in gastrointestinal tumour omics research

**Figure 2:**
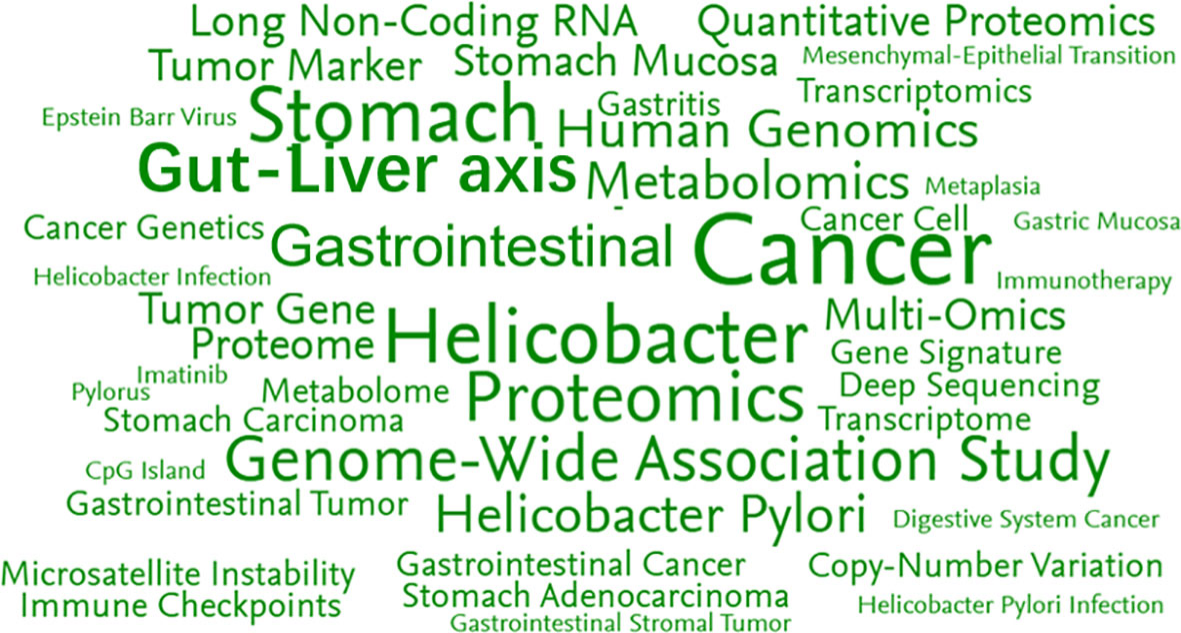
Top 50 key phrases by relevance, based on 1,382 publications.

To discern developments in gastrointestinal tumour omics research, we conducted a bibliometric analysis. A total of 3,116 omics-related publications on gastrointestinal tumours were retrieved from PubMed and Scopus databases, with keywords analysed across 1,382 papers spanning 2014 to 2026, as shown in **Figure 2**. Analysis of current rapid-emerging topics in omics revealed gastrointestinal tumours as the most prominent keyword, followed by metabolomics, proteomics, and transcriptomics research. This underscores the prominence of gene expression studies in recent years. Research targeting specific pathogens, such as the relationship between Helicobacter pylori and gastrointestinal tumours, highlights the impact of multi-omics approaches in disease prediction.

Summarising recent trends in popular omics methodologies reveals an explosive growth in long non-coding RNA (lncRNA) research, indicating lncRNA’s crucial role in elucidating gene regulation and cancer. Immunotherapy-related omics encompassing immune checkpoint inhibitors, tumour microenvironment analysis, and gene expression profiling represent prominent contemporary research avenues. Single-cell RNA sequencing analysis holds considerable promise for disease treatment. Exosome research plays a vital role in drug delivery; by integrating omics data with advanced imaging techniques—such as radiomics, computer-aided AI, tomography, and magnetic resonance imaging—it enhances disease treatment outcomes and improves survival rates.

### 2.2 Genomic insights into gastrointestinal tumours

Genomic research holds significant importance in understanding the heterogeneity of gastrointestinal tumours and identifying molecular subtypes of the disease.

Early large-scale sequencing projects have classified gastrointestinal tumours into distinct molecular subtypes (Sohn et al., 2017). Building upon this, Oh et al. further refined the classification into two major genomic subtypes—stromal and epithelial—through integrated genomic and proteomic studies, revealing substantial differences in genomic mutations and therapeutic responses between these subtypes (Oh et al., 2018). Similarly, Jeong et al. identified clinically conservative genomic subtypes in gastric adenocarcinoma, correlating them with patient prognosis and treatment efficacy (Jeong et al., 2023). These findings demonstrate that gastrointestinal tumours are not a single disease entity but comprise multiple genomic subtypes at the genetic level, with certain subtypes exhibiting greater aggressiveness and poorer prognosis.

A major direction in gastrointestinal cancer genomics research concerns the role of genomic instability in tumour progression. Huang et al. investigated the progression from high-risk intestinal metaplasia to gastrointestinal tumours, identifying genomic signatures serving as early biomarkers for cancer risk stratification (Huang et al., 2018). Relatedly, Zhang et al. focused on genomic instability-associated long non-coding RNAs (lncRNAs), developing a seven-lncRNA biomarker panel capable of predicting patient prognosis and enhancing immunotherapy efficacy (Zhang et al., 2023). These studies reveal how accumulated mutations and structural variations drive cancer formation, demonstrating that genomic instability correlates with disease prognosis.

Advances in precision treatment for gastrointestinal tumours are intrinsically linked to the deep application of genomic sequencing technologies: the VIKTORY trial by Lee et al. confirmed that comprehensive genomic sequencing of metastatic gastrointestinal tumours directly guides patients towards matched targeted therapies, significantly enhancing clinical efficacy (Lee et al., 2019); Cai et al. expanded the catalogue of driver mutations through large-scale gastrointestinal tumour genome sequencing, providing a comprehensive mutation landscape for targeted therapy decision-making (Lee et al., 2019). Furthermore, genomic analysis has identified therapeutically relevant genetic events: Kim et al. discovered that MTAP gene loss in gastrointestinal tumours correlates closely with tumour growth and serves as a biomarker for immunotherapy response (Kim et al., 2011). Collectively, these studies demonstrate that genomic data is evolving from guiding existing targeted drug applications towards identifying novel genomic vulnerabilities, progressively translating into personalised treatment strategies.

Comparative genomic analyses of molecular subtypes in gastrointestinal tumours elucidate the mechanisms underpinning their behavioural differences: Díaz-Serrano et al. identified that synergistic activation of the PI3K-AKT-mTOR pathway in HER2-positive gastrointestinal tumours significantly correlates with prognosis following trastuzumab therapy (Díaz-Serrano et al., 2018). Complementarily, Hu et al. identified mutation signatures specific to HER2-positive and -negative subtypes by comparing their genomic alterations, thereby proposing targeted personalised treatment strategies (Hu et al., 2023). Concurrently, Nakano et al. observed frequent PD-L1 gene amplification and JAK2 mutations in EBV-positive gastrointestinal tumours, alterations associated with favourable immunotherapy responses (Nakano et al., 2021). These findings underscore the importance of refined genomic classification based on HER2 status or EBV association, beyond broad subtype categorisation, for optimising treatment decisions and prognosis.

Continuous advances in sequencing technologies have significantly deepened genomic research in gastrointestinal tumours: Yu et al. revealed unique mutation patterns in Chinese patients through ultra-deep targeted sequencing, confirming the substantial clinical value of tumour genomic alteration profiles for treatment guidance (Yu P. et al., 2021); Xu et al. analysed the genomic characteristics of epithelial-mesenchymal transition (EMT) in gastrointestinal tumours, identifying potential targets for metastasis prevention (Xu et al., 2021); In metastatic disease research, Li et al. traced genomic evolution to elucidate mechanisms where specific alterations drive dissemination (Lee et al., 2023), while Oh et al. identified key genetic alterations in bone-metastatic gastrointestinal tumours, providing rationale for targeting metastasis-specific vulnerabilities (Oh et al., 2024). Yamamoto et al. systematically documented major driver mutations and gene amplifications through real-world genomic analysis, proposing novel therapeutic targets for specific patient cohorts (Yamamoto et al., 2024).

The clinical application of genomics in risk stratification and novel diagnostics for gastrointestinal tumours demonstrates direct translational value: Cheong et al. developed and validated a 32-gene expression signature based on large-scale genomic data, which defines prognostic subtypes and predicts chemotherapy response, laying the foundation for clinical genomic risk scoring (Cheong et al., 2022); Kanda et al. demonstrated the suitability of routine formalin-fixed paraffin-embedded specimens for next-generation sequencing, with their standardised workflow establishing the feasibility of genomic testing within routine clinical practice (Kanda et al., 2023); Chen et al. identified characteristic genomic alterations in cerebrospinal fluid ctDNA analysis of patients with meningeal metastases from gastrointestinal tumours, providing biomarkers for non-invasive monitoring (Chen et al., 2023). These studies converge upon a core consensus: integrating genomics into clinical decision-making—encompassing early detection, prognostic assessment, and treatment guidance—constitutes the pivotal pathway for advancing the field.

### 2.3 Transcriptomic insights into gastrointestinal tumours

Transcriptomic studies of gastrointestinal tumours analyse whole-transcriptome expression profiles to elucidate gene regulatory mechanisms, identify molecular subtypes, and uncover clinically relevant biomarkers. Cristescu et al. classified gastrointestinal tumours into four subtypes based on gene expression patterns: mesenchymal-like, microsatellite instability, TP53-active, and TP53-inactive. each associated with distinct molecular alterations and prognosis. The mesenchymal subtype, characterised by enriched epithelial-mesenchymal transition and stem cell features, carries poor prognosis, highlighting the value of expression signatures in patient risk stratification (Cristescu et al., 2015). Furthermore, Chan et al. investigated the RNA editing enzymes ADAR1 and ADAR2, revealing widespread dysregulation of RNA editing in nearly all tumours. ADAR1 exhibits oncogenic effects while ADAR2 possesses tumour-suppressing properties, with their editing activities influencing transcripts associated with tumour progression. This indicates that RNA editing profiles may serve as potential biomarkers for disease progression (Chan et al., 2016); Concurrently, Szász et al. integrated a large-scale transcriptomic database of 1,065 patients, systematically validating survival-associated genes (e.g., high expression of BECN1, CASP3, CTNNB1, and SIRT1 predicts favourable prognosis, whereas BIRC5, TIMP1, MMP2, and VEGF indicate poor prognosis), thereby establishing a validation platform for prognostic mRNA biomarkers (Szász et al., 2016). Collectively, traditional batch transcriptomics studies have elucidated molecular subtypes and key regulatory pathways (such as the Wnt/β-catenin pathway), laying the groundwork for hypothesis-driven exploration of novel targets.

The advent of single-cell RNA sequencing (scRNA-seq) technology signifies a transformative breakthrough in transcriptomics. Its capacity to resolve gene expression at single-cell resolution fundamentally alters the averaging approach to tumour heterogeneity employed in traditional batch analyses: Zhang’s team constructed a single-cell atlas spanning over 30,000 cells from normal gastric tissue to early gastrointestinal tumours (Zhang P. et al., 2019), tracking carcinogenic progression and identifying novel markers such as OR51E1—a molecule specifically recognising unique endocrine cell subpopulations in early tumours. Furthermore, Li’s team discovered that ARID1A (a chromatin remodeller) mutations enhance expression of immune checkpoint molecules like PD-L1, thereby shaping an immunogenic tumour microenvironment (Li et al., 2019). These studies not only elucidate the cellular and molecular mechanisms underpinning gastrointestinal tumour development but also provide novel targets for precision medicine strategies.

Single-cell transcriptomics has demonstrated critical utility in deciphering the tumour microenvironment (TME) of gastrointestinal cancers: Wang et al. identified unique transcriptional states among tumour cell subpopulations through single-cell sequencing of metastatic gastric adenocarcinoma, thereby deriving a 12-gene signature significantly correlated with survival outcomes (Wang et al., 2021); Kumar et al. constructed a multi-type single-cell atlas of gastrointestinal tumours encompassing immune and stromal cells, revealing elevated plasma cell proportions as a distinguishing marker between diffuse and intestinal tumours (Kumar et al., 2022); Li et al. further discovered specific enrichment of Tregs within the TME of gastrointestinal tumours alongside active immune suppression programmes (Li et al., 2022). Collectively, these studies illuminate the profound intratumoural heterogeneity and dynamic cellular interaction networks within gastrointestinal tumours, offering novel perspectives for precision therapeutic strategies.

Spatial transcriptomics, as a crucial complement to single-cell RNA sequencing, deciphers gene expression while preserving spatial information: Tsubosaka et al. integrated single-cell and spatial technologies to construct gastric maps, revealing interaction mechanisms between epithelial and stromal cells (e.g., fibroblasts) in normal versus neoplastic tissues (Tsubosaka et al., 2023); Shi et al. developed the SpatialTME integrated platform, merging histological and transcriptomic data to visualise the spatial arrangement of immune and tumour cells, thereby revealing associations between specific immune cell clusters and tumour progression or therapeutic response (Shi et al., 2024); Dong et al. performed single-cell analysis on paired primary-metastatic gastrointestinal tumour samples, identifying significant differences in immune microenvironment composition (e.g., altered T cell/macrophage ratios in metastatic sites) (Dong et al., 2024). These studies underscore the pivotal role of tumour spatial positioning and contextual environment, suggesting therapeutic strategies must incorporate spatial heterogeneity considerations.

### 2.4 Proteomic insights into gastrointestinal tumours

Proteomics offers a breakthrough perspective for gastrointestinal tumour research by comprehensively analysing proteins—the core executors of cellular functions—transcending the limitations of single-dimensional genomic/transcriptomic studies. Early investigations focused on identifying differentially expressed proteins between tumour and normal tissues. For instance, Ryu et al. successfully identified gastric tumour-specific protein spots through two-dimensional electrophoresis (2-DE) separation of gastrointestinal tumour tissue proteins (Ryu et al., 2003). With advances in 2-DE coupled with mass spectrometry, numerous tumour-specific proteins associated with various gastrointestinal cancers have been validated. These studies not only reveal distinctive proteomic signatures of gastrointestinal tumours but also directly demonstrate systemic disruptions in their metabolic and translational pathways.

Innovations in mass spectrometry sensitivity and throughput have significantly propelled gastrointestinal tumour proteomics: Mohri et al. utilised SELDI-TOF mass spectrometry to compare tumour, adjacent non-tumour tissue, and serum samples, identifying candidate biomarkers including macrophage migration inhibitory factor (MIF) and human neutrophil peptides (HNP1-3) (Mohri et al., 2009); Kang et al. employed high-resolution mass spectrometry to record differentially abundant proteins, establishing a serum biomarker screening system (Kang et al., 2016); Zhou et al. further extended this to plasma proteomics, revealing the potential of fibrinogen alpha chain and apolipoprotein A-II/C-I expression alterations as early diagnostic markers (Zhou et al., 2020); More instructively, Osório et al. discovered that diabetic co-morbidity remodels the proteomic profile of gastrointestinal tumours (e.g., altering expression patterns of proteins associated with oxidative stress and metabolism) (Osório et al., 2021). Collectively, these studies broaden the dimensions for discovering biomarkers in gastrointestinal tumours.

The integration of proteomics with genomics/transcriptomics is profoundly reshaping our understanding of gastrointestinal tumour mechanisms: Zhang’s team utilised proteomic profiling to reveal that reduced m6A RNA methylation levels drive tumour progression by activating oncogenic pathways such as Wnt and PI3K-AKT (Zhang C. et al., 2019); Jiang et al., utilising immunoproteomics, identified specific protein signatures of chromosomal instability and immune evasion in TP53-mutant gastrointestinal tumours (Jiang et al., 2018); Ge et al. integrated multi-omics data to classify diffuse gastrointestinal tumours into three molecular subtypes (each possessing distinct protein networks and mutation profiles), identifying subtype-specific immunotherapy targets (Ge et al., 2018). These studies not only establish causal links from DNA alterations to functional protein outputs but also provide theoretical foundations for precision classification and treatment.

Proteomics research in gastrointestinal tumours continues yielding pivotal discoveries: Mun et al. classified early-stage tumours into four molecular subtypes by integrating proteomic and genomic features (Mun et al., 2019); Chen et al. revealed metformin’s anti-cancer mechanism through regulating cancer-associated fibroblast proteomics—involving CALML3 upregulation to inhibit tumour growth (Chen et al., 2019). This field has transitioned from single-marker identification to multidimensional omics integration: innovations in mass spectrometry sensitivity and algorithms are driving clinically valuable breakthroughs (e.g., kinase target discovery, protein-based stratified diagnostics), though challenges such as data standardisation and dynamic range remain to be overcome (Kang et al., 2016; Osório et al., 2021). These advances mark a pivotal leap in the translation of gastrointestinal tumour proteomics towards precision medicine.

### 2.5 Metabolomic insights into gastrointestinal tumours

Metabolomics, through the systematic analysis of biological metabolites—instantaneous snapshots of cellular physiological states—has emerged as a pivotal field for elucidating the mechanisms driving the malignant phenotype of gastrointestinal tumours. Early studies revealed that lipid and amino acid metabolic profiles in gastrointestinal tumour patients distinctly differ from those in non-cancerous populations (Gao et al., 2021; Yu L. et al., 2021): Yu et al. precisely identified biomarkers associated with lipid metabolic disorders through serum non-targeted metabolomics (Yu L. et al., 2021); Wang et al. integrated metabolomics and lipidomics strategies to characterise a distinctive metabolite-lipid signature capable of distinguishing early-stage gastrointestinal tumours from healthy individuals (Wang et al., 2024). These studies not only confirm the central role of metabolic reprogramming in gastrointestinal tumour development but also offer novel perspectives for early diagnosis.

Metabolomics demonstrates formidable potential in elucidating gastrointestinal tumour metastasis mechanisms and advancing non-invasive diagnostics: Chen et al. identified significant metabolite differences between metastatic and non-metastatic gastric tumours (Chen et al., 2010); Pan et al. further characterised unique metabolic fingerprints in peritoneal fluid from patients with peritoneal metastasis (Pan et al., 2020); At the diagnostic application level, Chen et al. (2024) constructed a machine learning model based on ten plasma metabolites to achieve precise differentiation and survival prediction for gastrointestinal tumour patients (Chen et al., 2024); Quan et al. confirmed that urinary metabolites can serve as an early screening tool for gastrointestinal tumours (Kwon et al., 2020). These breakthroughs—alongside successful validation of metabolomics in gastrointestinal diseases such as coeliac disease (Upadhyay et al., 2020)—are collectively establishing a multi-tiered diagnostic framework for gastrointestinal tumours, driving innovation in therapeutic strategies.

Metabolomics research has revealed dysregulation in core metabolic pathways such as aminoacyl-tRNA synthesis and lipid reprogramming within gastrointestinal tumours (Gao et al., 2021; Wang et al., 2024). Future efforts must focus on establishing standardised, multi-centre protocols and validating metabolite diagnostic models through large-scale cohort studies (Chen et al., 2024; Qu et al., 2024). These advances are profoundly expanding our understanding of the biochemical mechanisms underlying gastrointestinal tumours, pioneering new dimensions for precision intervention.

### 2.6 Single-cell omics insights into gastrointestinal tumours

Single-cell omics technologies (including scRNA-seq and single-cell DNA sequencing) resolve gastrointestinal tumour cell heterogeneity at revolutionary resolution, profoundly elucidating clonal evolution, therapeutic resistance, and immune evasion mechanisms: Zhao et al. integrated conventional transcriptomics with single-cell sequencing to precisely identify prognosis-associated gene clusters—markers often overlooked in low-resolution analyses—and further characterised their associated therapeutic targets (Zhao et al., 2021); Jia et al. elucidated complex immune interaction networks through single-cell sequencing of B cells and tertiary lymphoid structures in gastrointestinal tumour tissues (Jia et al., 2021); novel prognostic models constructed from such data surpass the limitations of traditional batch sequencing (Wang et al., 2021; Hu et al., 2022; Kumar et al., 2022; Jia et al., 2024). These breakthroughs not only unlock functional insights into rare cellular subpopulations but also reshape the precision diagnosis and treatment paradigm for gastrointestinal tumours.

Single-cell technologies are reshaping the research paradigm for gastrointestinal tumour treatment response: Zheng et al. integrated single-cell and transcriptome data to establish a ‘personalised stem cell-related’ gene signature, enabling precise prediction of potential responders to therapies targeting stem cell-like cells (Zheng et al., 2024); Chen et al. (2022) employed longitudinal single-cell sequencing before and after neoadjuvant chemotherapy to dynamically reconstruct the treatment-driven tumour microenvironment remodelling process for the first time (Chen et al., 2022), such studies not only provide functional blueprints for identifying resistance markers but also pioneer new dimensions for temporal therapeutic strategies.

Single-cell omics technology decodes core mechanisms of immune evasion in gastrointestinal tumours: Ji et al. identified TAGLN2-specific upregulation in malignant epithelial cells driving peritoneal metastasis (Ji et al., 2023), revealing how heterogeneous expression of immune checkpoint molecules (e.g., PD-L1/PD-1) across tumour subclones shapes immune-privileged microenvironments; More critically, this technology provides the first direct evidence that macrophages induce stem-like gastrointestinal tumour cell development through metabolic reprogramming (Sung and Cheong, 2022). These high-resolution insights not only refine prognostic models and biomarker systems but also establish tumour-microenvironment interaction networks as a new therapeutic intervention axis.

### 2.7 Multi-omics insights into gastrointestinal tumours

Integrated multi-omics technologies overcome the limitations of single-dimensional understanding in gastrointestinal tumours by fusing multi-dimensional genomic, epigenomic, transcriptomic, proteomic, and metabolomic data. This approach reveals novel tumour characteristics unattainable through individual omics studies (Argelaguet et al., 2018; Nam et al., 2021): Liu et al. (2024) elucidated a novel mechanism whereby the Helicobacter pylori effector YWHAE drives gastrointestinal tumours by regulating the ferroptosis axis (Liu et al., 2024); Xu et al. (2022) constructed an unsupervised deep learning model integrating transcriptomic and epigenomic data to define novel molecular subpopulations exhibiting significant survival disparities (Xu et al., 2022); At the clinical translation level, Liu et al. (2021) developed a precision risk scoring system based on a copy number variation-gene expression-methylation composite biomarker (Liu et al., 2021); Ma et al. (2024) further integrated transcriptomic-proteomic-metabolomic data to pioneer the Mitochondrial Function-Centred Prognostic Model (MitoScore) (Ma et al., 2024). Collectively, these studies have laid the foundation for a multimodal diagnostic and therapeutic framework for gastrointestinal tumours.

### 2.8 Omics insights into the roles of macrophages and fibroblasts in gastrointestinal tumours

Within the gastrointestinal tumour microenvironment, macrophages and cancer-associated fibroblasts constitute a pivotal regulatory hub—through dynamic equilibrium with tumour cells and each other, they shape an ecological niche exhibiting both pro-tumour and anti-tumour properties (Katoh and Nakagama, 2014; Sung and Cheong, 2022).

Within the gastrointestinal tumour microenvironment, the plasticity of tumour-associated macrophages (TAMs) drives critical pathological processes: hypoxia inhibits glycolysis via the microRNA-30c/mTOR axis and induces M2 polarisation (Zhihua et al., 2019), while DGAT1-mediated lipid metabolic reprogramming significantly accelerates tumour progression (He et al., 2021). Single-cell sequencing further reveals functional heterogeneity among TAM subpopulations—including the ‘metastasis-promoting’ M2-like subset dominating malignant ascites (Eum et al., 2020), the CD47+ subset possessing immune-editing capabilities (Shi et al., 2021), and machine-learning-identified subtypes harbouring unique oncogenic drivers (e.g., CAST) (Wei et al., 2020; Wang et al., 2021; Hwang et al., 2022; Yang et al., 2022). Collectively, these findings construct a dual metabolic-immune regulatory network for TAMs, establishing novel intervention axes for targeting the tumour microenvironment.

Cancer-associated fibroblasts (CAFs), as the dominant force in the gastrointestinal tumour stroma, shape malignant progression through extracellular matrix remodelling, inflammatory regulation, and paracrine signalling networks: Shen et al. discovered that dedifferentiated CAFs drive cancer cell proliferation by upregulating caveolin-1 (Shen et al., 2015), while SPARC expression deficiency emerged as a key negative regulator enhancing chemotherapy resistance and tumour stemness (Ma et al., 2019). Gu et al. integrated multi-omics data via machine learning to establish an activity-based CAF subtyping system, enabling precise stratification of gastrointestinal tumour stroma characteristics (Gu et al., 2023) (Ma et al., 2019). Gu et al. employed machine learning to integrate multi-omics data, establishing a CAF activity phenotyping system enabling precise stratification of gastrointestinal tumour stroma characteristics (Gu et al., 2023). At the molecular interaction level, Hong et al. utilised spatial transcriptomics combined with single-cell sequencing to reveal the TGF-β1-IGFBP7 paracrine circuit inducing diffuse gastrointestinal tumour infiltration phenotypes (Hong et al., 2023), while Lee et al. (2024) demonstrated that fibroblast-derived TINAGL1 directly promotes tumour progression via integrin β1 interactions (Lee et al., 2024). Collectively, these findings delineate a multidimensional regulatory framework for CAFs, pioneering novel therapeutic paradigms targeting the tumour stroma.

The synergistic interaction between macrophages and cancer-associated fibroblasts (CAFs) is reshaping the evolutionary paradigm of gastrointestinal tumour microenvironments: Li et al. (2023) revealed that IGFBP7 secreted by CAFs under cancer cell stimulation recruits and polarises macrophages via the FGF2/FGFR1/PI3K/AKT axis (Li D. et al., 2023); Jiang et al. (2024) further uncovered antagonistic competition between these cells centred on nicotinamide metabolism—bidirectionally regulating the activation state of both the stroma and cancer cells by modulating NAD⁺ bioavailability (Jiang et al., 2024). These breakthrough discoveries, grounded in multi-omics integration, directly establish three novel therapeutic axes: targeting macrophage functional remodelling, blocking key secretory factors of CAFs, and disrupting metabolic symbiosis networks.

### 2.9 Interactions between the gut microbiome and gastric cancer/liver disease from a multi-omics perspective

The gut microbiome has emerged as a molecular hub within the gastric cancer-liver disease interaction network: its dysbiosis drives gastric carcinogenesis and accelerates liver disease progression (e.g., cirrhosis) through immune-inflammatory cascade activation, metabolic reprogramming, and toxic signalling via the gut-liver axis (Lazăr et al., 2025; Mohamed et al., 2025). In the context of liver disease, dysbiosis and barrier disruption in the gut facilitate the translocation of microorganisms and their products—such as pathogen-associated molecular patterns (PAMPs)—to the liver via the portal vein. These signals activate innate immune pathways in the liver (such as Toll-like receptors [TLRs] and inflammasomes), reprogram the state of Kupffer cells and stellate cells, amplify cytokine/chemokine cascades, and thereby systemically regulate the gastrointestinal mucosal microenvironment and tumour progression. Concurrently, microbe-driven metabolic remodelling—including bile acid conversion, short-chain fatty acid utilisation, and altered tryptophan/indole metabolic flux—simultaneously accelerates hepatic inflammatory fibrosis and gastrointestinal carcinogenesis by modulating epithelial signalling, mucosal immune homeostasis, and oxidative stress responses. Crucially, the microbiome functions as a mechanistic “master switch” in this comorbidity, revealed through multi-omics integration: metagenomics and metatranscriptomics capture functional activity at the strain level; metabolomics quantifies bile acids and microbial metabolites; host transcriptomics/proteomics decipher immune and barrier phenotypes in tumour and liver tissues; while single-cell and spatial omics unravel cell-communication networks shaped by microbial products (e.g., myeloid-epithelial and stroma-immune interactions). This multidimensional integration reconstructs causal pathways from dysbiosis to immune editing, metabolic vulnerability, therapeutic response, and organ crosstalk, establishing a systems biology foundation for precision diagnosis and treatment of co-morbidity.

Multi-omics integration (genomics → transcriptomics → proteomics → metabolomics) systematically decodes microbiota-mediated mutation profiles, biomarker networks, and metabolically vulnerable targets; while AI-empowered machine learning engines achieve breakthroughs in uncovering cross-omics correlations, enabling dynamic disease prediction and optimising personalised treatment paradigms (Maan et al., 2023). Machine learning algorithms effectively integrate data across omics levels to uncover latent connections between gut microbiota, gastric cancer, and liver disease (Morabito et al., 2025). AI models identify key biomarkers from multi-omics datasets, predict disease onset and progression, and guide personalised therapies. These synergistic advances collectively establish a new era of precision diagnostics and therapeutics targeting the microbiome.

## 3 Discussion

Gastrointestinal tumour omics research is undergoing a revolution in the deep integration of six-dimensional genomic data (genome-transcriptome-proteome-metabolome-single-cell-spatial omics). By correlating mutation profiles with downstream transcriptional/protein dynamics (Oh et al., 2018; Wang et al., 2021), researchers have for the first time reconstructed the causal chain linking DNA variations to functional phenotypes. The fusion of spatial transcriptomics and single-cell sequencing enables more precise mapping of the spatiotemporal evolution of tumour subclones within the microenvironment (Tsubosaka et al., 2023; Shi et al., 2024). These breakthroughs not only drive iterative upgrades to molecular subtyping systems but also decode core carcinogenic pathways *in situ* within tissues, ultimately establishing a new framework for ‘multi-omics-driven diagnostics and therapeutics’.

Multidimensional breakthroughs have emerged in gastrointestinal tumour omics research: transcriptomics (including single-cell) unlocks non-coding RNA regulatory axes and key biomarkers (Cristescu et al., 2015; Chan et al., 2016; Xu et al., 2017); proteomics, aided by mass spectrometry, precisely maps dynamic signalling pathways and post-translational modification networks (Kang et al., 2016); while metabolomics systematically reveals conserved tumour vulnerability targets such as lipid/amino acid metabolism (Gao et al., 2021; Wang et al., 2024)—with AI-driven multimodal data fusion engines significantly enhancing interpretative depth (Liu et al., 2021; Xu et al., 2022). Nevertheless, three core challenges persist in this field: research quality heterogeneity necessitates independent validation consortia; retrospective designs dominate, urgently requiring prospective clinical trial translation; and cross-platform data barriers impede meta-analyses. The solution lies in establishing a multi-omics hub database for gastrointestinal tumours, integrating genomic, transcriptomic, proteomic, metabolomic, and clinical data. This will usher in a new era for validation studies in the field.

Targeted microbiome interventions represent a core strategy for comorbidities management, requiring balanced consideration of antitumour effects and hepatic safety. This includes optimising dietary patterns (e.g., high-fiber/bile acid-modulating diets), applying targeted prebiotics/probiotics/synbiotics and postbiotics to restore barrier function and immune-metabolic homeostasis, and employing narrow-spectrum antimicrobials when necessary. For refractory dysbiosis or severe microecological translocation phenotypes, microbiota transplantation or rational microbiota therapy may be employed under rigorous screening. Emerging technologies such as phage therapy, engineered bacterial delivery systems, and microbial enzyme small-molecule inhibitors are advancing precision interventions. Multi-omics integration (metagenomics/metabolomics/host transcriptomics) enables patient stratification to guide combined treatment decisions—e.g., selecting immunotherapy combinations based on microbial immunomodulatory profiles, matching metabolic-targeted therapies to populations with bile acid/lipid reprogramming advantages, and optimising chemotherapy regimens based on liver reserve function and gut-mediated toxicity prediction. To achieve clinical translation, we established a four-step workflow: (1) baseline assessment of liver disease severity and tumour staging; (2) development of combined microbiota-metabolite biomarkers for risk stratification; (3) creation of algorithmic models integrating multi-omics and clinical variables to recommend microecology-antitumour combination regimens; (4) dynamic monitoring for treatment adjustment and complication prevention. Prospective studies must incorporate standardised sample collection, liver disease-specific safety endpoints, and defined microbiota/metabolite subgroups to validate clinical utility.

## 4 Conclusion

Nevertheless, translating these omics research findings into clinical practice remains fraught with significant challenges. A primary obstacle lies in integrating vast multidimensional datasets and interpreting them as actionable information for clinical decision-making. Advanced artificial intelligence and machine learning techniques require further refinement to automate and standardise the analysis of patient omics data, while concurrently developing clinical decision support tools. Another challenge lies in ensuring omics technologies and their benefits reach populations globally, particularly in regions with high disease prevalence where resources for comprehensive genomic sequencing may be lacking (Karimi et al., 2014).

To combat gastrointestinal cancers more effectively worldwide, it is imperative to facilitate the dissemination of advanced omics technologies and knowledge to regions with limited research capacity. Balancing contributions to global gastrointestinal cancer research will enrich data diversity and ensure conclusions possess broad applicability. Advancements in gastrointestinal oncology omics will also benefit from broader cancer research ecosystems—including breakthroughs in immuno-oncology, targeted therapies, and computational biology—while reciprocally informing these fields.

The field of gastrointestinal oncology omics is undergoing a technology-driven cognitive revolution: genomics enables precision molecular subtyping; transcriptomics (including single-cell techniques) provides multidimensional analysis of subtype heterogeneity, regulatory RNA networks, and novel biomarkers; Proteomics and metabolomics synergistically decode aberrant signalling pathways and identify metabolically vulnerable targets; while AI-enabled integrative analysis breakthroughs in mapping dynamic tumour microenvironment topologies, *in situ* elucidating co-evolutionary mechanisms of heterogeneity and therapeutic resistance—collectively reshaping the framework for gastrointestinal cancer disease analysis and establishing a new paradigm for precision diagnostics and therapeutics.

Future strategies for gastrointestinal oncology omics research must focus on five core pillars: establishing gold-standard integrated workflows for genomic-epigenomic-transcriptomic-proteomic-metabolomic data to drive cross-study reproducibility; strategically investing in AI-enabled bioinformatics infrastructure to intelligently extract biological logic from complex datasets; pushing the boundaries of single-cell/spatial omics/organoid modelling to *in situ* decipher early evolutionary trajectories of gastrointestinal tumours; establishing multinational, multi-centre validation consortia to accelerate translation of discoveries within heterogeneous populations; ultimately closing the loop from ‘omics discovery to clinical pathways’—optimising early diagnosis through biomarker combinations, developing therapeutic decision algorithms, and prospectively validating targeted strategies to pioneer a new era of precision diagnostics and therapeutics for gastrointestinal tumours.

By implementing this strategic pathway, the gastrointestinal oncology research community will reshape the landscape of precision oncology: through deep integration of multi-dimensional omics data with clinical insights—enabling molecular mechanism-guided ultra-early diagnosis, dynamic optimisation of personalised therapies, and precision monitoring of recurrence—ultimately establishing a diagnostic-therapeutic closed loop centred on patient survival benefit.
